# Supplementary material for: Individual differences in frontoparietal plasticity in humans
Source: NPJ Sci Learn. 2022 Jun 23;7:14. doi: 10.1038/s41539-022-00130-1 (PMC9226021; doi:10.1038/s41539-022-00130-1)
Supplement: Supplementary file 1 — Supplemental Material [file 41539_2022_130_MOESM1_ESM.pdf]

Supplementary Files

	Pre-Training M (SD)	***Post-Training M (SD)***	***Change M (SD)***
**VTA rsFC**			
Left LPFC	.12 (.17)	.08 (.19)	-.04 (.23)
Right LPFC	.21 (.16)	.23 (.17)	.02 (.21)
MPFC	.10 (.17)	.11 (.18)	.01 (.24)
Parietal	.12 (.17)	.13 (.18)	.01 (.23)
Striatum	.20 (.18)	.18 (.18)	-.02 (.24)
**T1w/T2w Ratio**			
Left LPFC	.94 (.13)	.93 (.12)	-.01 (.03)
Right LPFC	.91 (.13)	.91 (.13)	-.001 (.04)
MPFC	.89 (.12)	.88 (.12)	-.01 (.04)
Parietal	.96 (.13)	.05 (.12)	-.01 (.04)
Striatum	1.10 (.13)	1.09 (.13)	-.01 (.03)
**2-back > 1-back Betas**			
Left LPFC	27.12 (18.10)	21.60 (19.92)	-5.52 (28.27)
Right LPFC	28.19 (19.69)	20.60 (25.68)	-7.59 (31.89)
MPFC	19.33 (15.51)	15.21 (20.01)	-4.11 (24.22)
Parietal	29.35 (21.33)	27.24 (24.81)	-2.11 (28.18)
Striatum	9.48 (8.85)	4.76 (11.41)	**-4.72* (14.01)**

Supplementary Table 1. Means and standard deviations of neural measures at baseline and following training, as well as training-related changes in these features. Bold values with an asterisk denote significant training-related changes, as indicated by Student's *t*-test. * = $p \leq 0.05$. Abbreviations: lateral prefrontal cortex (LPFC), medial prefrontal cortex (MPFC), resting-state functional connectivity between the ventral tegmental area and frontoparietal regions of interest (VTA rsFC).

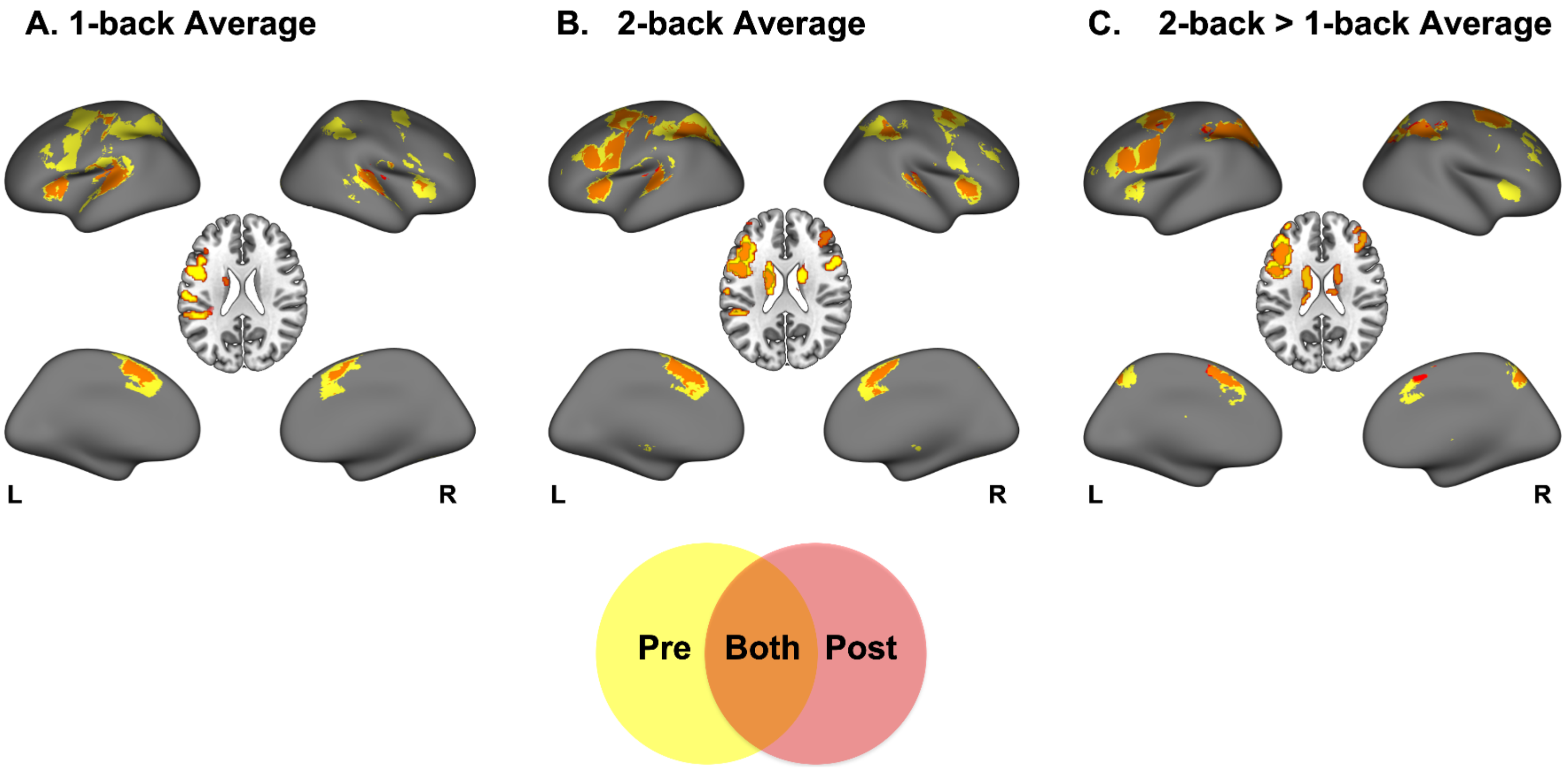

Supplementary Figure 1. *N*-back task activation before and after adaptive *n*-back practice. Average functional activation for (A) 1-back > baseline, (B) 2-back > baseline, and (C) 2-back > 1-back. Activation at baseline (pre-training) is shown in yellow, activation post-training is shown in red, and their overlap is shown in orange. Pre-training regions in the 2-back > 1-back contrast (shown in yellow in Panel C) were used as regions of interest in the VTA functional connectivity and T1w/T2w ratio analyses. Results are corrected for multiple comparisons at $z = 4.0$, $p < 0.05$. Axial slices are shown for $z = 22$.

A. Left LPFC

	Δ VTA rsFC	Δ T1w/T2w	Δ Betas
Δ T1w/T2w	-.10		
Δ Betas	.002	-9.452e-05	
Δ ACC	-2.492e-02	.09	-.0003
Δ RT	.03	-.25	-.001

B. Right LPFC

	Δ VTA rsFC	Δ T1w/T2w	Δ Betas
Δ T1w/T2w	-.70		
Δ Betas	.001	-7.317e-05	
Δ ACC	-.04	.01	-1.284e-04
Δ RT	.002	-.27	-.0004

C. MPFC

	Δ VTA rsFC	Δ T1w/T2w	Δ Betas
Δ T1w/T2w	-.12		
Δ Betas	-.0002	-.0003	
Δ ACC	.04	.10	-9.699e-05
Δ RT	-.03	.02	-.0001

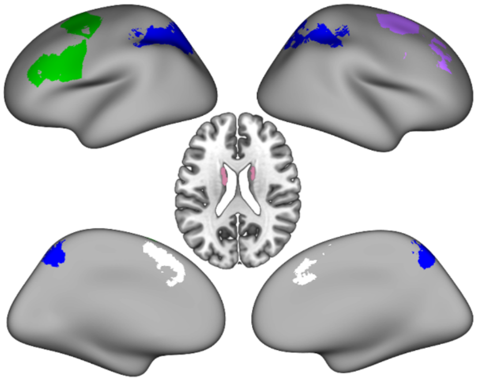

D. Parietal

	Δ VTA rsFC	Δ T1w/T2w	Δ Betas
Δ T1w/T2w	-9.095e-02		
Δ Betas	-.001	-.0002	
Δ ACC	1.987e-04	.09	-.0003
Δ RT	.04	.01	-.001

E. Striatum

	Δ VTA rsFC	Δ T1w/T2w	Δ Betas
Δ T1w/T2w	-1.04		
Δ Betas	.003	-.001	
Δ ACC	.02	.04	-9.928e05
Δ RT	-.01	-.43	-.001

Supplementary Figure 2. Beta coefficients for regression models examining associations between training-related changes in neural measures and working memory performance, rounded to the nearest hundredth. Axial slice visualized at $Z = 22$. Abbreviations: lateral prefrontal cortex (LPFC), medial prefrontal cortex (MPFC), resting-state functional connectivity between the ventral tegmental area and frontoparietal regions of interest (VTA rsFC), T1w/T2w ratio "myelin map" values (T1w/T2w), 2-back > 1-back beta values (Betas), *n*-back accuracy (ACC), *n*-back response times (RT). Statistical models control for age, sex, baseline working memory performance, baseline brain measures, and motion.
