# Supplementary material for: Individual differences in frontoparietal plasticity in humans
Source: NPJ Sci Learn. 2022 Jun 23;7:14. doi: 10.1038/s41539-022-00130-1 (PMC9226021; doi:10.1038/s41539-022-00130-1)
Supplement: Supplementary file 2 — Reporting Summary Checklist [file 41539_2022_130_MOESM2_ESM.pdf]

nature portfolio

Corresponding author(s): Austin Boroshok, MA

Last updated by author(s): May 26, 2022

Reporting Summary

Nature Portfolio wishes to improve the reproducibility of the work that we publish. This form provides structure for consistency and transparency in reporting. For further information on Nature Portfolio policies, see our Editorial Policies and the Editorial Policy Checklist.

Statistics

For all statistical analyses, confirm that the following items are present in the figure legend, table legend, main text, or Methods section.

n/a	Confirmed	
☐	☒	The exact sample size (*n*) for each experimental group/condition, given as a discrete number and unit of measurement
☐	☒	A statement on whether measurements were taken from distinct samples or whether the same sample was measured repeatedly
☐	☒	The statistical test(s) used AND whether they are one- or two-sided<br>*Only common tests should be described solely by name; describe more complex techniques in the Methods section.*
☐	☒	A description of all covariates tested
☐	☒	A description of any assumptions or corrections, such as tests of normality and adjustment for multiple comparisons
☐	☒	A full description of the statistical parameters including central tendency (e.g. means) or other basic estimates (e.g. regression coefficient) AND variation (e.g. standard deviation) or associated estimates of uncertainty (e.g. confidence intervals)
☒	☐	For null hypothesis testing, the test statistic (e.g. *F*, *t*, *r*) with confidence intervals, effect sizes, degrees of freedom and *P* value noted<br>*Give P values as exact values whenever suitable.*
☒	☐	For Bayesian analysis, information on the choice of priors and Markov chain Monte Carlo settings
☒	☐	For hierarchical and complex designs, identification of the appropriate level for tests and full reporting of outcomes
☐	☒	Estimates of effect sizes (e.g. Cohen's *d*, Pearson's *r*), indicating how they were calculated

Our web collection on statistics for biologists contains articles on many of the points above.

Software and code

Policy information about availability of computer code

Data collection	All behavioral data was collected using custom MATLAB scripts (Version: MATLAB 9.4 R2018a).
Data analysis	All analyses were conducted in R (R version 4.05; RStudio Version 1.4.1106).

For manuscripts utilizing custom algorithms or software that are central to the research but not yet described in published literature, software must be made available to editors and reviewers. We strongly encourage code deposition in a community repository (e.g. GitHub). See the Nature Portfolio guidelines for submitting code & software for further information.

Data

Policy information about availability of data

All manuscripts must include a data availability statement. This statement should provide the following information, where applicable:

- Accession codes, unique identifiers, or web links for publicly available datasets
- A description of any restrictions on data availability
- For clinical datasets or third party data, please ensure that the statement adheres to our policy

All behavioral data, task-based and control regions of interest (ROIs), values extracted from neuroimaging data are freely available at https://github.com/austinboroshok/frontoparietal-plasticity. Deidentified neuroimaging data in BIDS format are freely available at https://openneuro.org/datasets/ds003849/versions/1.0.0. All code used to collect functional task data and to analyze behavioral and imaging data are freely available at https://github.com/austinboroshok/frontoparietal-plasticity.

Human research participants

Policy information about studies involving human research participants and Sex and Gender in Research.

Reporting on sex and gender	Participants reported their biological sex. Recruitment and analyses did not differ by sex. Sex was included as a covariate in all analyses. Consent for sharing data was obtained from all participants.
Population characteristics	See below
Recruitment	The study participants were recruited through the University of Pennsylvania study recruitment system, as well as through community and university advertisements. Use of this largely undergraduate and graduate student sample may limit generalizable of learning findings to the broader population.
Ethics oversight	This study was approved by the University of Pennsylvania's Institutional Review Board. Written informed consent was obtained from all participants.

Note that full information on the approval of the study protocol must also be provided in the manuscript.

Field-specific reporting

Please select the one below that is the best fit for your research. If you are not sure, read the appropriate sections before making your selection.

☐ Life sciences ☒ Behavioural & social sciences ☐ Ecological, evolutionary & environmental sciences

For a reference copy of the document with all sections, see nature.com/documents/nr-reporting-summary-flat.pdf

Behavioural & social sciences study design

All studies must disclose on these points even when the disclosure is negative.

Study description	Quantitative cross-sectional
Research sample	Sample composition: University of Pennsylvania undergraduate and graduate students (95%), Philadelphia community members (5%)<br>Age: Participants were between 18 and 25 years old (Mean: 21.39 years, SD = 1.91 years)<br>Sex: 63% female<br><br>The sample is not representative and was collected via convenience sampling. Variability in learning in college students in essential to understand and provides a useful model for approximating neuroplasticity in adults.
Sampling strategy	The study participants were recruited via convenience sampling through the University of Pennsylvania study recruitment system, as well as through community and university advertisements. This sample size met a predetermined target set by a power analysis indicating that such a sample size would have 80% power to detect a correlation between brain measures and learning of r = 0.4.
Data collection	The 50-minute working memory training period, as well as the pre- and post-training assessments, were administered on a laptop. Participants were given 2000 ms to respond via button press on a standard keyboard: "F" for "YES" responses and "J" for "NO" responses. For the in-scanner n-back task, responses were recorded using a standard button box. Researchers were not blind to the study hypotheses during data collection.
Timing	Data collection began in April 2017 and was completed in February 2019.
Data exclusions	Inclusion criteria included fluency in English, no history of psychiatric or neurological disorders or learning disabilities, no current or recent substance use, and no contraindications for MRI.<br><br>Participants were excluded for falling asleep during the n-back scan (n = 3), low performance on the control condition of the fMRI task (<90% accuracy on the 1-back condition; n = 5), failure to advance beyond the initial working memory condition of 2-back during the 50-minute training period (n = 1), substance use (not reported during screening but reported during participation; n = 1), inability to tolerate scanning (n = 1), and technical issues (total n = 4 button box malfunction [n = 2], coil error [n = 1], no behavioral log files [n = 1]).
Non-participation	No participants dropped out or declined to participate.
Randomization	Our study design did not include randomization. Age and sex were included as covariates in all analyses.

Reporting for specific materials, systems and methods

We require information from authors about some types of materials, experimental systems and methods used in many studies. Here, indicate whether each material, system or method listed is relevant to your study. If you are not sure if a list item applies to your research, read the appropriate section before selecting a response.

Materials & experimental systems

n/a	Involved in the study
☒	☐ Antibodies
☒	☐ Eukaryotic cell lines
☒	☐ Palaeontology and archaeology
☒	☐ Animals and other organisms
☒	☐ Clinical data
☒	☐ Dual use research of concern

Methods

n/a	Involved in the study
☒	☐ ChIP-seq
☒	☐ Flow cytometry
☐	☒ MRI-based neuroimaging

Magnetic resonance imaging

Experimental design

Design type

Task (block design), resting-state

Design specifications

Out-of-scanner n-back task:
The out-of-scanner task consisted of four blocks of trials at each of 3 cognitive conditions (2-, 3- and 4-back, alternating in that order) for a total of 12 blocks. Each of the 12 blocks contained 24 trials, which lasted 2500 ms each. Each block was followed by 10 seconds of rest.

n-back Training
The 50-minute n-back training session began at the 2-back condition. Each block at each condition consisted of 24 trials. Participants completed a mean of 745 trials (31.1 blocks), each of which lasted 2500 ms. Intervals between trials varied as the training period was self-paced. Participants progressed to the next-highest task condition if they finished blocks at or above 90% accuracy, remained at the same task condition if they finished blocks with 71-89% accuracy, and regressed to the next lowest condition (min: 2-back) if they finished blocks with 70% accuracy or below.

In-scanner n-back task:
The in-scanner n-back task consisted of four 30-second blocks alternating between two conditions (1- and 2-back), with 12 trials per block. Each of these blocks were followed by 10 seconds of rest.

Behavioral performance measures

We measured learning, or improved performance on the n-back test, using two separate indices: (1) accuracy, defined as the change in the percentage of correctly-answered trials following training, and (2) response time, defined as the change in response time across trials following training.

To establish that participants were performing the task as expected, we excluded participants whose mean accuracy on the 1-back condition of the pre-training n-back task was below 90% correct.

Acquisition

Imaging type(s)

Functional, structural

Field strength

3T

Sequence & imaging parameters

During both the pre- and post-training scan sessions, participants completed an identical series of scans:

(1) One whole-brain, high-resolution, T1-weighted multi-echo scan (MEMPRAGE, TR = 2530 ms; TEs = 1.69, 3.55, 5.41, 7.27 ms; flip angle = 7°; resolution = 1 mm isotropic)

(2) One T2-weighted structural scan (T2SPACE, TR = 3200 ms; TE = 406 ms; resolution = 1 mm isotropic; turbo factor: 282), collected with volumetric navigators (Tisdall et al., 2012)

(3) One five-minute resting-state fMRI scan (TR = 2000ms; TEs = 30.20 ms; flip angle = 90°; resolution = 2 mm isotropic)

(4) One five-minute scan of an n-back fMRI task (TR = 2000 ms, TE = 30.2 ms, flip angle = 90°, voxel size = 2.0 x 2.0 x 2.0 mm, matrix size = 96 x 96 x 75, 75 axial slices, 170 volumes, field of view = 192 mm)

Area of acquisition

Whole brain scans were collected.

Diffusion MRI ☐ Used ☒ Not used

Preprocessing

Preprocessing software	Preprocessing for the task-based fMRI data was implemented using FEAT (FMRI Expert Analysis Tool) Version 6.00, part of FSL (FMRIB's Software Library, www.fmrib.ox.ac.uk/fsl). The following steps were applied: motion correction using MCFLIRT (Jenkinson et al., 2002), skull stripping, spatial smoothing using a Gaussian kernel of FWHM 5 mm, and high-pass temporal filtering (100s).<br><br>Resting state data were preprocessed with a different pipeline than the one used for the task-based data, in order to incorporate Nipype, a Python-based framework specifically optimized for flexibly integrating resting-state analysis tools (Gorgolewski et al., 2011). The software packages used in this preprocessing pipeline included FMRIB Software Library (FSL v5.0.8; Jenkinson et al., 2012), FreeSurfer (v6.0; Dale et al., 1999), Advanced Normalization Tools (ANTs v2.1.0; Avants et al., 2011), and Nipype's implementation of Artifact Detection Tools (ART; http://www.nitrc.org/projects/artifact_detect/). Simultaneous realignment and slice timing correction was conducted using an algorithm implemented in Nipy (Roche, 2011). The functional data were bandpass filtered (0.01–0.1 Hz) and spatially smoothed with an isotropic 6 mm Gaussian kernel (FWHM).
Normalization	The task data were normalized to the MNI template during a two-step process using FLIRT (FMRIB's Linear Image Registration Tool) in FEAT.<br><br>The resting-state data were normalized to the OASIS-30 Atropos template (in MNI152 2 mm space) using Freesurfer's bbregister and ANTs.
Normalization template	Task data were normalized to the MNI template.<br><br>Resting-state data were normalized to the OASIS-30 Atropos template in MNI152 2 mm space.
Noise and artifact removal	For task analyses, motion was corrected during preprocessing using MCFLIRT, and first- and group-level general linear models (GLM) for each participant included FSL's standard and extended motion parameters (global signal, 6 motion parameters and their temporal derivatives, quadratic terms, and the temporal derivatives of the quadratic terms) as regressors.<br><br>For resting-state analyses, outlier volumes in the functional resting-state data (based on composite motion [>0.5 mm of head displacement between volumes] and global signal intensity [>3 SD from the mean]) were discarded using ART. At the single-subject level, outlier volumes flagged by ART (one nuisance regressor per outlier) and composite motion were regressed out. Five principal components were derived from CSF and white matter segmentations and regressed from the resting-state data, in order to correct for physiological noise like heart rate and respiration (aCompCor; Behzadi et al., 2007).
Volume censoring	For both EPI sequences (n-back task and resting-state), the first four volumes of each scan were automatically discarded to allow time for scanner magnetization to reach equilibrium.

Statistical modeling & inference

Model type and settings	Task models were univariate. 1 run per participant at pre- and post-training timepoints. Group-level analyses were mixed effects analyses in FEAT (FLAME 1).
Effect(s) tested	We tested three contrasts of interest (1-back greater than baseline; 2-back greater than baseline; 2-back > 1-back) for Pre-training-only, Post-training-only, and Pre-training > Post-training.

Specify type of analysis: ☐ Whole brain ☒ ROI-based ☐ Both

Anatomical location(s)	To identify task-active ROIs, we used the results of the whole-brain analysis of the 2-back > 1-back contrast from the pre-training scan at a threshold of z = 4.0. We selected the five most significant clusters (Figure 2): (1) left lateral prefrontal cortex (left LPFC; MNI coordinates for center-of-gravity: X = -37 , Y = 10 , Z = 41), (2) right lateral prefrontal cortex (right LPFC; MNI coordinates for center-of-gravity: X = 29, Y = 12, Z = 52), (3) bilateral medial prefrontal cortex (mPFC; MNI coordinates for center-of-gravity: X = -1, Y = 16, Z = 47), (4) bilateral parietal cortex (including medial parietal regions; MNI coordinates for center-of-gravity: X = -4, Y = -56, Z = 48,), and (5) striatum (MNI coordinates for center-of-gravity: X = -7, Y = 3, Z = 12). To examine whether FPS regions specifically predict learning, we also examined two control regions: primary visual cortex and primary motor cortex. We defined these ROIs using the pericalcarine and precentral gyrus regions of the Harvard-Oxford probabilistic cortical structural atlas provided through FSL.<br><br>For resting-state functional connectivity analyses, the ventral tegmental area (VTA ROI was defined using a probabilistic atlas (Murty et al., 2014).

Statistic type for inference (See Eklund et al. 2016)	Cluster-wise: z > 4.0, corrected cluster significance threshold p = .05
Correction	FDR

Models & analysis

n/a	Involved in the study
☐	☒ Functional and/or effective connectivity
☒	☐ Graph analysis
☒	☐ Multivariate modeling or predictive analysis

Functional and/or effective connectivity: Pearson correlation
